# Lithium-Ion-Sieve Hydrogel Based on Aluminum Doping with High Stretchability, Strong Adsorption Capacity and Low Dissolution Loss

**DOI:** 10.3390/gels10110710

**Published:** 2024-11-01

**Authors:** Yujie Zhang, Yang Wang, Le Guo, Chenzhengzhe Yan, Long Li, Shuyun Cui, Yujie Wang

**Affiliations:** 1 Petroleum Engineering College, Xi’an Shiyou University, Xi’an 710000, China; zyjzxcv1284892080@163.com (Y.Z.); guole0807@163.com (L.G.); 17369477644@163.com (L.L.); 2 New Energy College, Xi’an Shiyou University, Xi’an 710000, China; yanczz123@163.com (C.Y.); c18992951115@163.com (S.C.); wangyujie_0603@163.com (Y.W.)

**Keywords:** lithium-ion-sieve hydrogel, adsorption, mechanical character, solution loss

## Abstract

In recent years, with the development of the new energy industry, lithium resources need to be supplied in large quantities. The lithium-ion sieve (LIS) is regarded as an ideal adsorbent for recovering lithium resources from brine because of its excellent lithium adsorption capacity and structural stability. However, because it is powdery after molding, and there will be problems such as dissolution loss of manganese, which limits its industrial development. In this study, in the process of preparing hydrogels of acrylic acid (AA), acrylamide (AM) and chitosan (CS), an LIS hydrogel with high mechanical properties, strong adsorption capacity and low dissolution loss was prepared by doping LIS and Al ions. Among them, the stress of the prepared chitosan–acrylic acid–acrylamide hydrogel (PASA-1) with an Al doping content of 1% reached 603 KPa, and the maximum strain reached 189%, which showed excellent damage resistance. In addition, the adsorption performance of PASA-1 reached 43.2 mg/g, which was excellent, which was attributed to the addition of Al ions, which inhibited the dissolution loss of manganese ions. This idea has great potential in the direction of lithium resource recovery and provides a new method for the use of hydrogel in the direction of lithium-ion sieves.

## 1. Introduction

In recent years, because of the promulgation of the “double carbon” policy, the new energy industry has developed rapidly and vigorously. As an important part of this industry, lithium resources can be predicted to be consumed in large quantities [1]. Therefore, the task of developing lithium resources and finding substitutes for lithium resources is becoming increasingly important. Because most of the lithium resources in the world exist in salt lake brine, extracting lithium from salt lake brine has become an important direction of lithium resource production [2]. Up to now, the main technologies for extracting lithium from salt lake brine are precipitation [3], solvent extraction [4,5,6], membrane separation [7] and adsorption [8,9,10]. Among them, the adsorption method has become one of the most promising technologies for extracting lithium from salt lake brine with its advantages of high adsorption efficiency and low cost. The key of the adsorption method lies in the selection of adsorbents. Inorganic ion adsorbents have become the most promising adsorbents for their good selectivity and high adsorption efficiency [11]. The lithium-ion sieve (LIS) is a lithium-ion adsorbent with many excellent properties, such as low toxicity, low cost, high chemical stability and high lithium absorption capacity in industry. LIS is widely regarded as an adsorbent with great adsorption prospects in lithium-ion adsorption.

The lithium-ion sieve (LIS) is a typical example. As an adsorbent, LIS is not only easy to prepare and low in cost but is also environmentally friendly. Its unique pore structure and “ion sieveing” characteristics make it have an excellent adsorption effect and excellent selectivity for lithium ions. In particular, the theoretical adsorption capacity of the lithium-ion sieve H_1.6_Mn_1.6_O_4_ prepared by precursor Li_1.6_Mn_1.6_O_4_ can be as high as 72.3 mg/g [12]. Although the adsorption capacity of the LIS is excellent, its recovery effect is not good because it is powdery after molding, and this will lead to high manganese dissolution loss [13], which greatly limits its industrialization development process.

In order to overcome this problem, some scholars put forward that the lithium-ion sieve should be used as a device, such as in the granulation method [14], film forming method [15], foaming method [16] and doping method [17], to change the shape of the ion sieve, so as to put it into industrial use as soon as possible. However, after forming, the contact area with lithium ions is inevitably reduced, which reduces the adsorption sites, thus affecting the adsorption performance of lithium ions. Recently, researchers found that a modification can be carried out on the basis of the traditional membrane method, and a nano-fiber membrane with high porosity and a large contact area can be prepared by reacting LIS with polymers with special structures, which can successfully solve the problem of occupied adsorption sites. Cheng et al. [18] grafted 2-(hydroxymethyl)-12-crown-4-ether (2H12C4) with a large number of cavities on the surface of chitosan (CS) to prepare a chitosan nano-fiber membrane (CS-CE) modified by crown ether (CE). Its adsorption content can reach 297 mg/g in 1000 mg/L lithium-containing solution, and it also has excellent cycle stability. After five cycles, its adsorption content only drops to 267.3 mg/g. Although these modified membranes have good adsorption performance and good mechanical properties, their application range is limited because of their poor swelling rate and low toughness. Comparatively speaking, polymer reticulated hydrogels show a higher swelling rate, excellent flexibility and better mechanical properties.

Based on these advantages, relying on the advantages of low cost and a simple synthesis process, polymer reticulated hydrogels have been applied in various environments. For example, in the recovery of marine lithium resources, Xu et al. [19] prepared ion-sieve hydrogel to overcome the powder loss of extracting ions from seawater. However, due to its low expansion capacity and poor flexibility, it can not withstand the harsh marine environment for a long time, and its practical application is still limited [20]. Song Y et al. [21] prepared λ-MnO_2_@IG hydrogel, which has excellent lithium-ion adsorption performance, stronger than most adsorbents containing LIS. The more favorable direction is that porous hydrogels are easy to recover [22] and show ultra-stable cyclic lithium extraction performance, which is directly attributed to the further improvement of pore structure during the continuous regeneration of the hydrogels. The pore structure of ion-sieve hydrogel is further expanded, which shows that it has great application potential in complex marine environments [23]. Although the combination of LIS and hydrogel is successful, the Mn^3+^ contained in LIS will produce a Jahn–Teller effect and disproportionation reaction [24], resulting in the loss of manganese ions, which leads to a decrease in the lithium-ion adsorption content. Therefore, in order to ensure high adsorption efficiency in the adsorption process, it is necessary to explore how to inhibit the loss of manganese ions.

In this paper, a method of preparing lithium-ion-sieve hydrogel by doping LIS and Al ions into the hydrogel matrix of acrylic acid (AA), acrylamide (AM) and chitosan (CS) was studied. For Al ions, doping Al ions can not only enhance the mechanical properties of hydrogels through their metal bonds but also inhibit the dissolution loss of manganese ions. Specifically, because the radii of Al^3+^ and Mn^3+^ are close, and the bond energy of Al-O is higher than that of Mn-O, the valence of manganese will be improved, the structural stability will be enhanced and the Jahn–Teller effect will be suppressed. In addition, the gel can be prepared into any desired shape and size according to requirements, which effectively solves the problem that LIS powder is difficult to recycle. Finally, the prepared PASA-1 hydrogel achieved excellent adsorption performance, strong mechanical properties and low manganese dissolution loss. This work successfully provides an effective design method and analysis strategy for designing and preparing lithium-ion-sieve hydrogel with high destructive and stable adsorption performance.

## 2. Results and Discussion

### 2.1. Material Characterization Results

**Figure 1:**
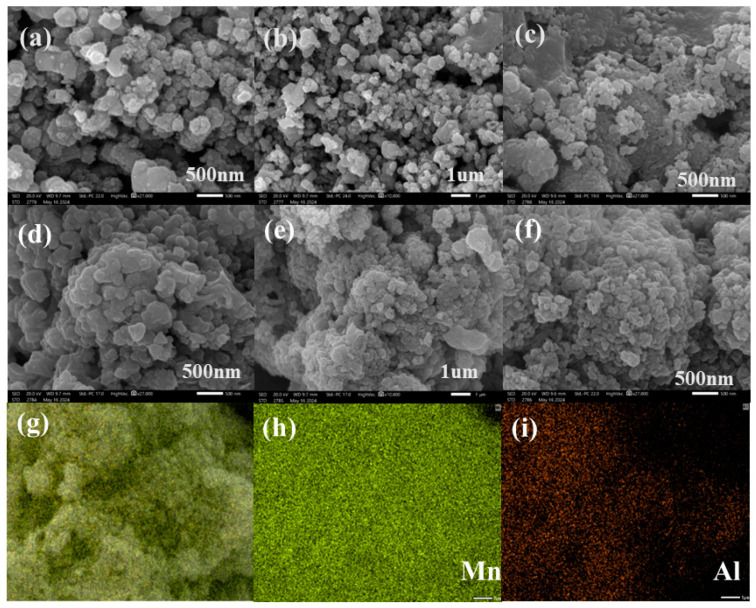
(**a**,**b**) SEM images of LMO under different magnifications, respectively; (**c**) SEM image of HMO; (**d**,**e**) SEM images of LMAO-4 under different magnifications; (**f**) SEM image of HMAO-4; (**g**–**i**) element mapping image of LMAO-4.

The appearance of the precursors LMO and LMOAO-4 before and after pickling was observed in detail. It can be clearly seen from Figure 1a,d that the synthesized precursors LMO and LMAO-4 are composed of polygonal particles with an average diameter of 200–400 nm, which are regular in shape but rough on the surface. For adsorbents, this rough surface helps to increase the void volume and specific surface area, which then improves the adsorption energy. However, after comparing Figure 1b,e, it can be seen that compared with LMO, the LMAO-4 samples doped with Al^3+^ ions show slight agglomeration. However, it is worth noting that despite the agglomeration, the diameter of the base particles has not changed obviously and remains in the range of 200–400 nm. This observation shows that the spinel crystal structure of the lithium-ion sieve has no obvious change after doping a small amount of Al^3+^ ions. In addition, Figure 1c,f further show that after acid treatment, the morphology of ion sieves HMO and HMAO-4 remains basically unchanged, and the crystal diameters are relatively uniform and close, which fully proves that the lithium–manganese lithium-ion sieves prepared in the experiment have excellent chemical stability and can resist the interference of external factors. In order to conduct an in-depth analysis of the element distribution of LMAO-4, this section uses energy-dispersion spectroscopy to test its energy spectrum in detail. The results are shown in Figure 1g–i. Through careful observation of these three images, it is clear that the Al element has been successfully and evenly incorporated into LMAO-4.

**Figure 2:**
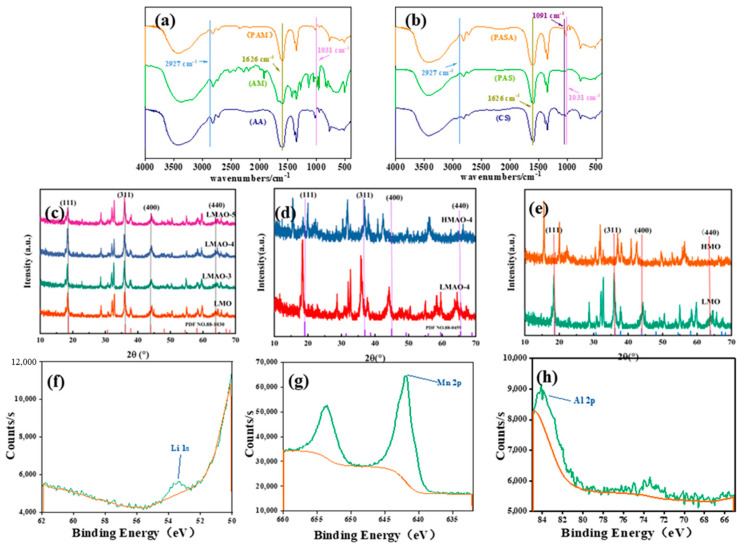
(**a**,**b**) Infrared spectra of different materials and hydrogels; (**c**) XRD patterns of LMO and Li_1.6_Mn_1.6__−__x_Al_x_O_4_; (**d**,**e**) XRD patterns of LMO and LMAO-4 after pickling; (**f**–**h**) the XPS spectra of Li, Mn and Al by HMAO.

As shown in Figure 2a, there is cross-linking of AA and AM in PAM hydrogels, and the vibrations of C-H (2927 cm^−1^), O-H (1626 cm^−1^) and C-O (1031 cm^−1^) in Fourier infrared spectra also appear on the PAM hydrogel curve. As shown in Figure 2b, there is AA, AM, and CS in PAS and PASA hydrogels, and the three are cross-linked with each other. The vibrations of C-H (2927 cm^−1^), N-H (1552 cm^−1^), O-H (1626 cm^−1^), C-N (1091 cm^−1^) and C-O (1031 cm^−1^) in Fourier infrared spectra also appear on the curves of PAS and PASA hydrogels. This shows that the prepared material exists in the hydrogel and its structure has not been damaged.

Figure 2c shows the comparison of the XRD spectra of doped Li_1.6_Mn_1.6__−__x_Al_x_O_4_ and undoped LMO. It can be concluded that there is no significant difference in the peak shape of LMAO samples doped with Al^3+^ ions compared with undoped LMO. The results show that the crystal structure of ion sieves is not changed after doping Al^3+^ ions, indicating that the doping modification is more successful. In order to further explore the effect of pickling on the crystal structure of the ion sieve, the XRD spectra of LMO, HMO and HMAO-4 were compared, such as in Figure 2d,e. It was observed that the diffraction angle of the peak of HMO shifted slightly to the left after acid leaching, and the peak shape did not change obviously, indicating that the structure of HMO after acid leaching was still the same as that before acid leaching. After doping with Al^3+^ ions, the characteristic peaks of HMAO-4 become sharper and clearer, with a complete peak shape. Compared with the standard spectrum of Li_1.6_Mn_1.6_O_4_, this observation fully proves that aluminum has been successfully doped into the lithium-ion sieve. For details of the XRD pattern of lithium-ion-sieve hydrogel, see Figure S7 of the supporting information. Figure 2f–h show peak types of Li, Mn, and Al; according to the comprehensive evaluation of XPS, the prepared LIS has very good structural stability. See Figure S8 of the supporting information for the details of other XPS spectra.

### 2.2. Mechanical Properties of Hydrogel

**Figure 3:**
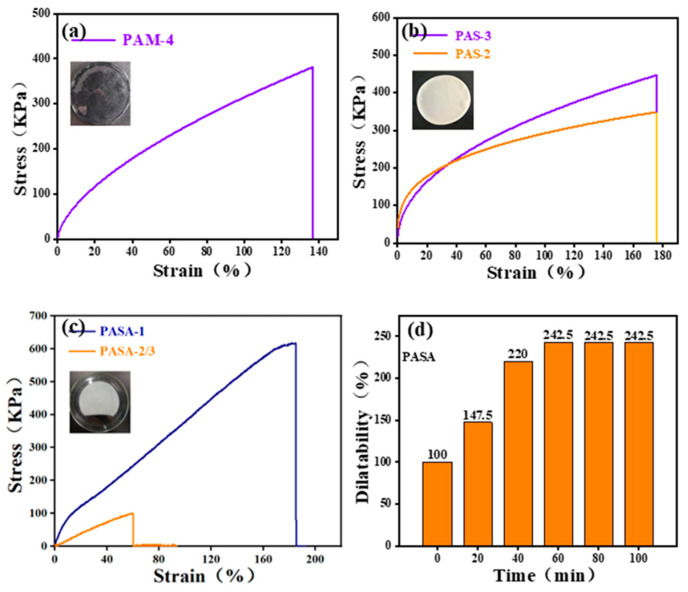
(**a**–**c**) The stress–strain curves of hydrogels of PAM, PAS and PASA with different proportions, and (**d**) the expansion rate histogram of PASA hydrogels.

In order to explore the influence of different acrylic acid contents on the mechanical properties of hydrogel, a series of experiments were carried out according to gradient ratio, and tensile tests were carried out to determine the physical and mechanical properties of the PAM hydrogel system. Due to the nature of acrylic acid itself, polymerization can occur. In order to study the influence of different proportions of acrylic acid and acrylamide on the mechanical properties of the hydrogel system, the uniaxial tensile test of hydrogel was carried out. As shown in Figure 3a, except for the sample with a 4:1 ratio, the performance is too low to obtain accurate test results. PAM-4 hydrogel can bear large stress, but the strain range of hydrogel is not large, which can reach 138% at the highest point. This may be due to the insufficient polymerization degree of the hydrogel itself and the weak cross-linking network of hydrogel. PAM hydrogels other than that in the 4:1 ratio may produce too many hydrogen bonds due to the presence of too many amide bonds in the polymer molecular chain, which leads to excessive cross-linking of the hydrogel and reduces the physical and mechanical properties of the hydrogel. However, with the further increase in the proportion of acrylic acid, the polymerization effect becomes worse, which seriously affects the mechanical properties of the hydrogel.

Due to the excellent properties of chitosan, it was studied whether the addition of chitosan could enhance the mechanical properties of the PAM hydrogel system and whether the addition of chitosan at different contents could affect the whole hydrogel system. Due to the hydrogen bonding between acrylic acid and chitosan, a special semi-interpenetrating cross-linked network was formed, and the mechanical properties of the hydrogel were improved to some extent. As shown in Figure 3b, compared with PAM hydrogel, PAS hydrogel can withstand greater stress without fracture, with stress increased by 16.3%, and the strain performance of hydrogel was also improved to a certain extent, up to 175%, with an increase of 26.8%. However, the other PAS hydrogels added with 1 g and 1.5 g chitosan may be due to the uneven cross-linking of hydrogels under the dual effects of physical and chemical cross-linking, or the excessive cross-linking of hydrogels, which makes the stress in hydrogels concentrated and leads to a decline in the physical and mechanical properties of hydrogels. With the increase in chitosan content in PAS hydrogel, the hardness of the hydrogel also increases, and the hydrogel has a certain brittleness, which makes it easy to break and may also reduce the mechanical properties of the hydrogel.

In order to study the effect of adding aluminum metal ions into the PAS hydrogel system on the physical and mechanical properties of the hydrogel, a tensile experiment of the prepared PAS hydrogel doped with Al was carried out. After the preliminary experiment of adding aluminum, the feasibility of preparing the hydrogel was determined. Due to the addition of metal bonds, the cross-linking network inside the hydrogel becomes more complicated, and the physical and mechanical properties of the hydrogel are improved to some extent. As shown in Figure 3c, the hardness of the hydrogel has been significantly improved due to the influence of the metal bond energy of the aluminum ion itself. When the aluminum content is 1%, the maximum stress of the PASA hydrogel can reach 603 KPa, and the maximum strain can reach 189%. Compared with PAA and PAS hydrogels, the stress and strain improves noticeably, by 36.9% and 6.2%, respectively. However, when the doping amount is 2% and 3% (the data gap is too small, which leads to the overlapping of images), the mechanical and physical properties of the hydrogel are greatly reduced. The possible reason is that when the metal ions are doped too much, the cross-linking network inside the hydrogel becomes excessive, which leads to stress concentration inside the hydrogel, thus reducing the mechanical properties of the hydrogel. Figure 3d shows the water absorption and swelling performance of PASA hydrogel, which can reach 242.5% at the highest point. In practical application, this hydrogel have increased surface area for treating a salt solution, which makes lithium ions enter the adsorption site more easily, thus improving the adsorption performance of the lithium-ion sieve. See Figure S9 of the supporting information for details of the swelling rate maps of other hydrogels.

### 2.3. Adsorption Performance of Lithium Ions

In order to evaluate the adsorption performance, LiCl solution containing 480 mg/L was prepared, and the pH value needed for the experiment was accurately adjusted by HCl and LiOH. At room temperature, the 0.1 g ion sieve was evenly mixed into 100 mL of this solution, followed by stirring with a magnetic stirrer at a speed of 350 r/min for two hours to ensure full reaction. After the reaction, the solid was carefully separated from the supernatant, and then the lithium-ion content in the solution after the reaction was accurately measured, so as to further analyze the adsorption efficiency of the ion sieve. The following formula can be used to calculate the lithium-ion adsorption performance:

(1) $$Q_{e}=\frac{V}{m}\left(C_{0}{-C}_{e}\right)$$

where Q_e_ is the lithium adsorption capacity of the adsorbent at equilibrium (mg/g), V is the volume of lithium solution (L), C_o_ is the content of Li^+^ in the initial lithium solution (mg/L), C_e_ is the content of Li^+^ in the solution at adsorption equilibrium (mg/L), and m is the mass of LIS (g).

**Figure 4:**
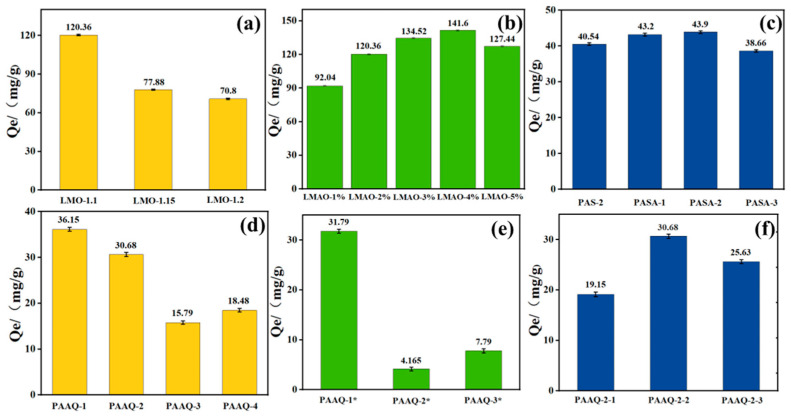
(**a**–**f**) Adsorption performance diagrams of LMO-X, LMAO-X, PASA hydrogel, PAAQ hydrogel, PAAQ-X* hydrogel, and PAAQ-2-X hydrogel, respectively.

Figure 4a,b show the adsorption performance of HMO on the Al^3+^ion-undoped lithium-ion sieve and HMAO on the Al-doped lithium-ion sieve with different doping levels. As the doping amount of Al^3+^ions increases, the adsorption performance of lithium-ion sieves shows a trend of first increasing and then decreasing. Except for the ion sieve with a doping amount of 1%, the adsorption performance of ion sieves with other doping amounts is higher than that of LIS doped with Al^3+^ions. This result shows that introducing Al element into the ion sieve can effectively improve its adsorption performance. This improvement is attributed to the successful doping of Al^3+^ions, which significantly reduces the Mn^3+^ content in Li_1.6_Mn_1.6__−__x_Al_x_O_4_. Due to the decrease in Mn^3+^content, the disproportionation reaction and Jahn–Teller effect induced by it are suppressed, thereby significantly enhancing the crystal structure stability and adsorption capacity of LIS. But when the doping amount exceeds a certain range, the adsorption capacity will decrease. This is mainly because excessive Al^3+^ can damage the spinel structure of the ion sieve, causing structural distortion and masking some adsorption sites. In addition, for ion sieves with only 1% doping, their adsorption performance is actually lower than that of undoped ion sieves. This may be due to the low doping level, resulting in an uneven distribution of Al ions in the ion sieve, which cannot fully exert its effectiveness, and they may even occupy the sites originally used for adsorption, thereby reducing the adsorption capacity.

From Figure 4c, it can be seen that when the content of hexahydrate aluminum chloride is 2%, LIS has the strongest ability to adsorb lithium ions. Compared with pure lithium-ion-sieve hydrogel, the adsorption capacity of lithium ions has been obviously improved. This may be due to the doping of metal ions, which enhances the original pore properties of lithium-ion-sieve hydrogels and provides more sites for lithium-ion adsorption, thus improving the exchange ability with lithium ions.

As can be seen from Figure 4d, the lithium-ion adsorption performance of the cellulose-doped lithium-ion-sieve hydrogel is obviously lower than that of the chitosan-doped lithium-ion-sieve hydrogel. This may be due to the compact structure of the hydrogel prepared from cellulose, which prevents lithium ions from entering the adsorption sites of lithium resources in the hydrogel from the solution during the adsorption test of lithium-ion-sieve hydrogel, thus reducing the adsorption performance of lithium resources. In order to test the influence of the cross-linking degree of hydrogel on adsorption performance, during the preparation of hydrogels, the degree of cross-linking of hydrogels was changed by controlling variables and adding MBA after other external conditions were unchanged; a series of PAAQ hydrogels were also prepared by changing the cross-linking time, as shown in Figure 4e,f. As can be seen from the figure, the adsorption properties of PAAQ lithium-ion-sieve hydrogels are further reduced with the increase in the cross-linking degree, and the adsorption properties of PAAQ-2* and PAAQ-3* are even more cliff-like. Similarly, under the conditions of different cross-linking times, the adsorption performance of PAAQ lithium-ion-sieve hydrogel with a cross-linking time of 4 h is the highest. PAAQ-13 may be formed due to the further improvement of the cross-linking degree, which leads to a decrease in the adsorption performance of the hydrogel, and PAAQ-11 may be formed due to a lower cross-linking degree of the hydrogel and the poor properties of the hydrogel itself, which leads to a decrease in adsorption performance.

**Figure 5:**
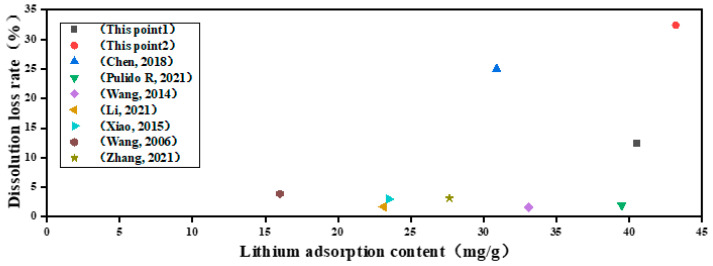
A comparison diagram of dissolution loss of lithium-ion sieves, where This point1 is HMAO-4, This point2 is HMO, and others are from [25,26,27,28,29,30,31].

Figure 5 shows a series of graphs showing the relationship between dissolution loss and the adsorption content of lithium-ion sieves, in which the prepared HMO shows an excellent adsorption capacity for lithium resources compared with others [25,26,27,28,29,30,31], but the relative dissolution loss rate is also high. In the improved HMAO-4, although the adsorption capacity for lithium resources is slightly reduced, the dissolution rate of the lithium-ion sieve is significantly reduced, which also further reduces cost in practice.

## 3. Conclusions

In this study, an effective design method and analysis strategy were successfully developed to design and prepare lithium-ion-sieve hydrogels with high destructive and strong adsorption properties. By optimizing the composition and preparation process of hydrogel, the adsorption capacity and mechanical strength of the lithium-ion sieve can be effectively adjusted. The stress and strain of PASA hydrogel doped with Al^3+^ can reach 603 KPa and 189%, respectively. This high mechanical performance fully protects the ion sieve from being damaged in complex environments. In addition, the PASA hydrogel with an Al^3+^ doping content of 1% exhibits an adsorption performance of 43.2 mg/g after being combined with the lithium-ion sieve. This fully proves that the doping of aluminum ions improves the adsorption performance and weakens the dissolution loss of manganese. This study not only provides a new material choice for the efficient recovery of lithium resources but also opens up a new road for the application of hydrogel in the direction of ion sieves.

## 4. Material and Methods

### 4.1. Material

Lithium chloride (LiCl specification AR), aluminum chloride hexahydrate (AlCl_3_∙6H_2_O specification 97.0%), lithium hydroxide monohydrate (LiOH∙H_2_O specification AR), manganese carbonate (MnCO_3_ specification ar), acrylic acid (specification > 99%), acrylamide (specification 99.0%), hydroxypropyl methylcellulose (HMPC), chitosan (specification ≥ 95%), N,N′-methylenebisacryamide (specification 99.0%), potassium persulfate (specification 97.0%), and all the above reagents were purchased from Aladdin Biochemical Co., Ltd. in Shanghai China. The water used in the experiment is pure water.

### 4.2. Preparation of Lithium-Ion Sieve

#### 4.2.1. Synthesis of Li_1.6_Mn_1.6_O_4_ Precursor

In order to prepare the required materials, 30 g of MnCO_3_ was first calcined in a muffle furnace at a high temperature of 800 °C for 5 h, and finally black Mn_2_O_3_ powder was obtained. Subsequently, according to different molar ratios of Li to Mn, an appropriate amount of Mn_2_O_3_ and LiOH∙H_2_O are accurately measured and fully ground in an agate mortar until they are completely fine and particle-free. After that, these finely mixed materials were moved to a hydrothermal reactor with a capacity of 100 mL, and the reaction continued at 120 °C for 48 h, thus generating LiMnO_2_. Finally, the prepared LiMnO_2_ was placed in the muffle furnace again and calcined at 450 °C for 4 h to obtain the final product Li_1.6_Mn_1.6_O_4_ [13]. According to different molar ratios of Li/Mn, these products were named LMO-1.1, LMO-1.15 and LMO-1.2, respectively.

#### 4.2.2. LIS Synthesis

Precursors with different ratios of lithium to manganese were placed in a beaker. According to the ratio of solid to liquid of 1:200, an appropriate amount of 0.5 mol/L HCl solution was placed in a magnetic stirrer and stirred at 25 °C for 24 h. After complete precipitation, the solid was eluted and filtered to neutrality, placed in an oven and dried at 80 °C for 6 h to obtain the corresponding H_1.6_Mn_1.6_O_4_ [13], which was named HMO-1.X. LIS doped with different contents of Al is referred to as LMAO-x%. For details, see Table S1 of the supporting information content and Figures S1 and S2.

### 4.3. Preparation of Hydrogel

**Figure 6:**
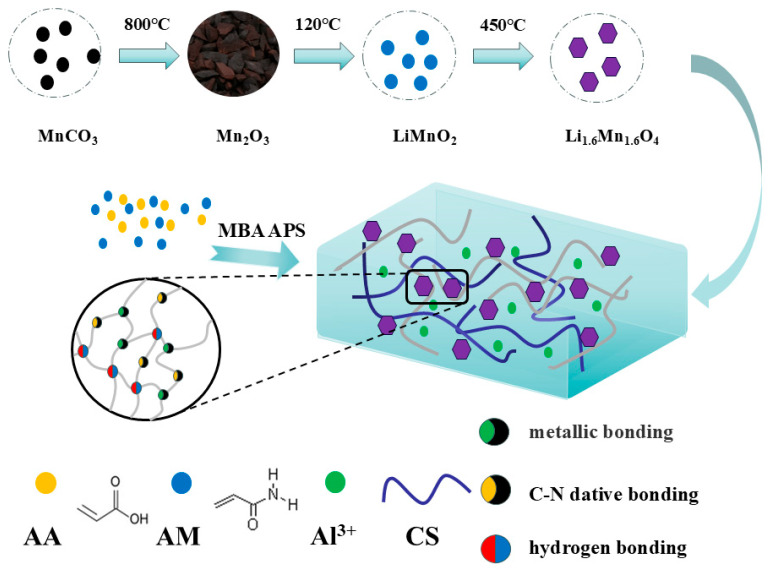
A schematic diagram of the manufacturing process of 1PASA’s lithium-ion-sieve hydrogel.

A series of hydrogels were prepared a by one-pot method to ensure the best scheme for the experiment. Firstly, prepare a series of gradient mixed solutions of acrylic acid and acrylamide. Add 0.04 g of 99% N,N′-methylenebisacrylamide and 0.39 g of 98% ammonium persulfate in sequence, and then place them in a collection-type constant-temperature heating stirrer until they are stirred for 4 h before removing. Place it in a liquid crystal ultrasonic cleaner and sonicate until the bubbles disappear. Then, place it in a mold in a vacuum drying oven at 60 °C for 4 h, remove it, and seal it in a bag. After selecting the optimal ratio, prepare a 3 wt% glacial acetic acid solution, weigh a quantitative amount of chitosan/x cellulose/LIS according to a series of ratios, and add it to the glacial acetic acid solution. Then, put it into a collection-type constant-temperature heating mixer until it is stirred evenly, and remove it. Repeat the above preparation steps and select the optimal ratio. (PAM is hydrogel of acrylic acid and acrylamide; PAS is PAM doped with CS; PASA is Al^3+^-doped PAS; PAAQ is a PAS hydrogel doped with HMPC.) See Tables S2–S6 and Figures S3–S6 in the Supporting Information. The overall preparation process is shown in Figure 6.

### 4.4. Characterization of Material Characteristics

A scanning electron microscope (SEM, model JSM7500F, made by JEOL) was used to observe the microstructure of the material in detail. In the sample preparation stage, a small amount of the samples was dissolved in ethanol at first, and the samples were uniformly dispersed by an ultrasonic wave. Subsequently, several drops of the dispersed sample solution were accurately sucked by using a pipette, and they were gently dripped on a copper net with a carbon film attached. After the ethanol was completely volatilized, the samples were observed and compared under the microscope.

The qualitative analysis of functional groups in materials using Fourier transform infrared spectroscopy (FTIR) is mainly based on the characteristic frequencies of infrared absorption spectra to identify which functional groups are present. Detect and analyze the structure of the material at the wavelengths of 500 to 4000; that is, use the information provided by the infrared absorption spectrum. Combine various properties of unknown substances and information provided by other structural analysis methods (such as ultraviolet absorption spectrum, nuclear magnetic resonance spectrum, mass spectrum) to determine the chemical structure or three-dimensional structure of the gel.

In order to deeply explore the internal structure of crystal materials, an X-ray powder diffractometer (model: D/MAX/2500PC, namely XRD) produced by Rigaku Company was adopted in this study. During the experiment, the Kα ray of Cu with the wavelength of 0.154056 nm was selected for the performance test. In order to ensure the stability of the experiment and the accuracy of the data, the test conditions can be set to voltage 40 kV and current 40 mA. In addition, in order to obtain high-resolution data, the scanning range was accurately controlled at a 2θ angle from 10 to 80, and the scanning step size was set at 0.02. After the test was completed, the scanned atlas was analyzed in detail by JADE software and compared with the standard PDF card one by one.

The surface of the sample was analyzed by X-ray photoelectron spectroscopy (XPS, Thermo Kalpha). Make sure that the sample is dry and tableted, and after the sample size reaches 3 mm × 3 mm and the thickness is less than 2 mm, test it, and finally obtain the peak shape and determine the element composition.

## Data Availability

The original contributions presented in this study are included in the article. Further inquiries can be directed to the corresponding author.

## Supplementary Materials

The following supporting information can be downloaded at https://www.mdpi.com/article/10.3390/gels10110710/s1, Figure S1: (a)–(e) LMAO-1, LMAO-2, LMAO-3, LMAO-4 and LMAO-5 in turn; Figure S2: (a)–(f) LMO-1.1, LMO-1.15, LMO-1.2, HMO-4, HMO-1.1 and Mn_2_O_3_ in turn; Figure S3: (a)–(e) PAM-1, PAM-2, PAM-3, PAM-4 and PAM-5 in turn; Figure S4: (a)–(c) PAS-1, PAS-2 and PAS-3 in turn; Figure S5: (a)–(c) PASA-1, PASA-2 and PASA-3 in sequence; Figure S6: (a)–(d) Lithium ion sieve hydrogels of PAS-2, PASA-1, PASA-2 and PASA-3; Figure S7: (a) shows the XRD pattern of LMO; (b) XRD pattern of LIS with lithium-manganese ratio of 1.15; (c)–(f) are XRD patterns of lithium ion sieve hydrogel doped with 0%-3%Al^3+^ respectively; Figure S8: (a) shows the XPS spectrum of LMAO when the ratio of lithium to manganese is 1.2; (b) XPS spectrum of element C in LMAO; Figure S9: (a)–(d) show the expansion rate maps of PAM, PAS, PAAQ, PAAQ-2-1 respectively; Table S1: Preparation Table of Lithium Ion Screen; Table S2: Preparation of Hydrogel Ingredients Table; Table S3: PAM hydrogel preparation table; Table S4: PAS hydrogel preparation table; Table S5: PASA hydrogel preparation table; Table S6: PAAQ hydrogel preparation table.
