# Supplementary material for: Lithium-Ion-Sieve Hydrogel Based on Aluminum Doping with High Stretchability, Strong Adsorption Capacity and Low Dissolution Loss
Source: Gels. 2024 Nov 1;10(11):710. doi: 10.3390/gels10110710 (PMC11594163; doi:10.3390/gels10110710)
Supplement: Supplementary file 1 [file gels-10-00710-s001.zip › gels-3261499-supplementary.pdf]

# Supporting information for Lithium-Ion-Sieve Hydrogel Based on Aluminum Doping with High Stretchability, Strong Adsorption Capacity and Low Dissolution Loss

**Yujie Zhang 1, Yang Wang 1,*, Le Guo 1, Chenzhengzhe Yan 2, Long Li 1, Shuyun Cui 2 and Yujie Wang 2**

**Table S1 Preparation Table of Lithium Ion Screen**

| Name | Li/Mn | $Al^{3+}$（%） |
|---|---|---|
| LMO-1.1 | 1 | × |
| LMO-1.15 | 1.15 | × |
| LMO-1.2 | 1.2 | × |
| LMAO-1 | 1 | 1 |
| LMAO-2 | 1 | 2 |
| LMAO-3 | 1 | 3 |
| LMAO-4 | 1 | 4 |
| LMAO-5 | 1 | 5 |

**Table S2 Preparation of Hydrogel Ingredients Table**

| Name | AA/AM | CS | Al | Cellulose | Degree of crosslinking | Time |
|---|---|---|---|---|---|---|
| PAM | √ | √ | × | × | × | × |
| PAS | √ | √ | × | × | × | × |
| PASA | √ | √ | √ | × | × | × |
| PAAQ | √ | √ | × | √ | × | × |
| PAAQ-X* | √ | √ | × | √ | √ | × |
| PAAQ-1X | √ | √ | × | √ | × | √ |

**Table S3 PAM hydrogel preparation table**

| Name | AA（g） | AM（g） | MBA（g） | APS（g） | Stress（KPa） | Strain（%） |
|---|---|---|---|---|---|---|
| PAM-5 | 2.5 | 0.5 | 0.04 | 0.39 | —— | —— |
| PAM-4 | 2 | 0.5 | 0.04 | 0.39 | 385 | 138 |
| PAM-3 | 1.5 | 0.5 | 0.04 | 0.39 | —— | —— |
| PAM-2 | 1 | 0.5 | 0.04 | 0.39 | —— | —— |
| PAM-1 | 0.5 | 0.5 | 0.04 | 0.39 | —— | —— |

**Table S4 PAS hydrogel preparation table**

| Name | CS（g） | Stress（KPa） | Strain（%） |
|---|---|---|---|
| PAS-3 | 1.5 | 448 | 175 |
| PAS-2 | 1 | 332 | 174 |
| PAS-1 | 0.5 | —— | —— |

**Table S5 PASA hydrogel preparation table**

| Name | $Al^{3+}$ | Stress（KPa） | Strain（%） |
|---|---|---|---|
| PASA-1 | 1% | 603 | 189 |
| PASA-2 | 2% | 84 | 96 |
| PASA-3 | 3% | 84 | 96 |

**Table S6 PAAQ hydrogel preparation table**

| Name | HMPC（g） | Experiment delayed joining MBA | Time（hour） |
|---|---|---|---|
| PAAQ-1 | 0.25 | × | 4 |
| PAAQ-2 | 0.5 | × | 4 |
| PAAQ-3 | 0.75 | × | 4 |
| PAAQ-4 | 1 | × | 4 |
| PAAQ-1* | 0.25 | √ | 4 |
| PAAQ-2* | 0.5 | √ | 4 |
| PAAQ-3* | 0.75 | √ | 4 |
| PAAQ-2-1 | 0.5 | × | 2 |
| PAAQ-2-2 | 0.5 | × | 4 |
| PAAQ-2-3 | 0.5 | × | 6 |

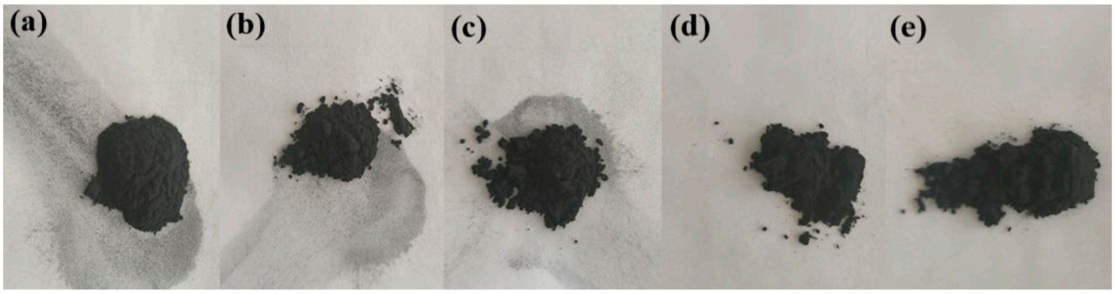


**Figure S1 (a)-(e) are LMAO-1, LMAO-2, LMAO-3, LMAO-4 and LMAO-5 in turn.**

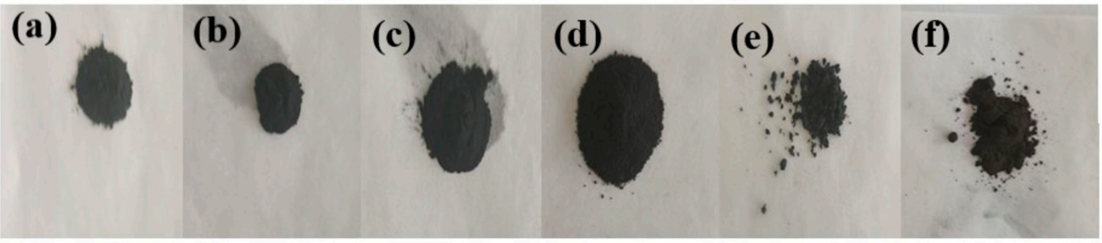


**Figure S2 (a)-(f) are LMO-1.1, LMO-1.15, LMO-1.2, HMO-4, HMO-1.1 and $Mn_2O_3$ in turn.**

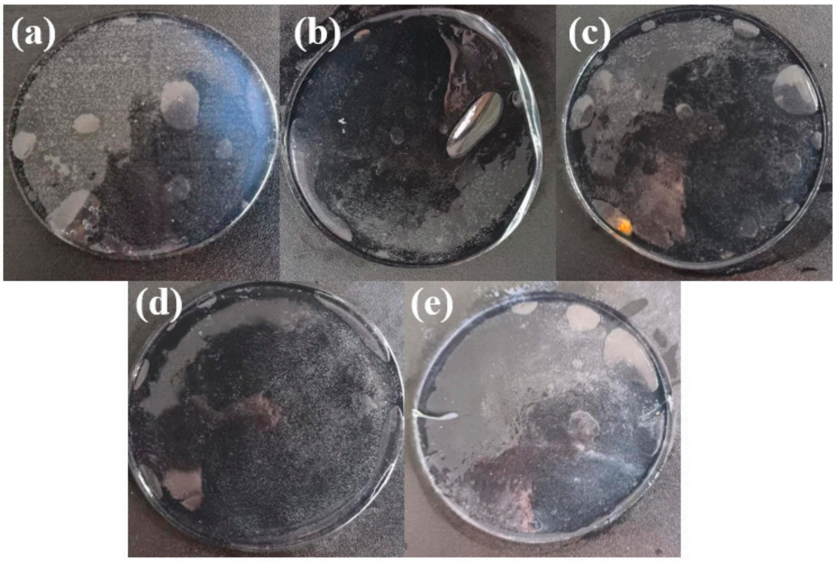


**Figure S3 (a)-(e) are PAM-1, PAM-2, PAM-3, PAM-4 and PAM-5 in turn.**

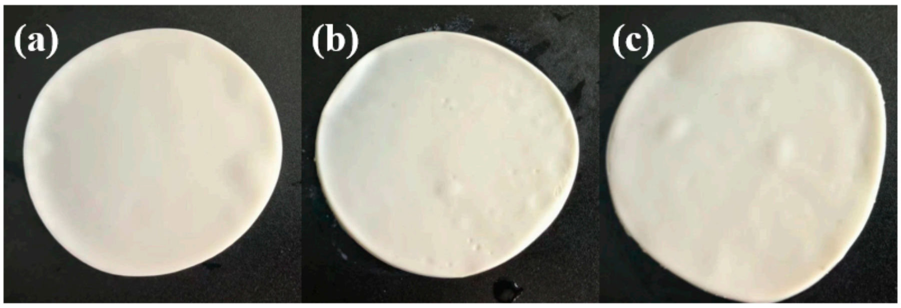


**Figure S4 (a)-(c) are PAS-1, PAS-2 and PAS-3 in turn.**

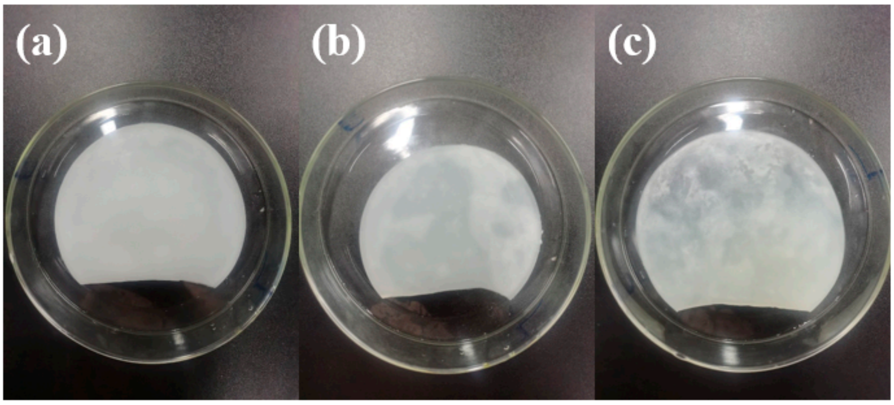


**Figure S5 (a)-(c) shows PASA-1, PASA-2 and PASA-3 in sequence.**

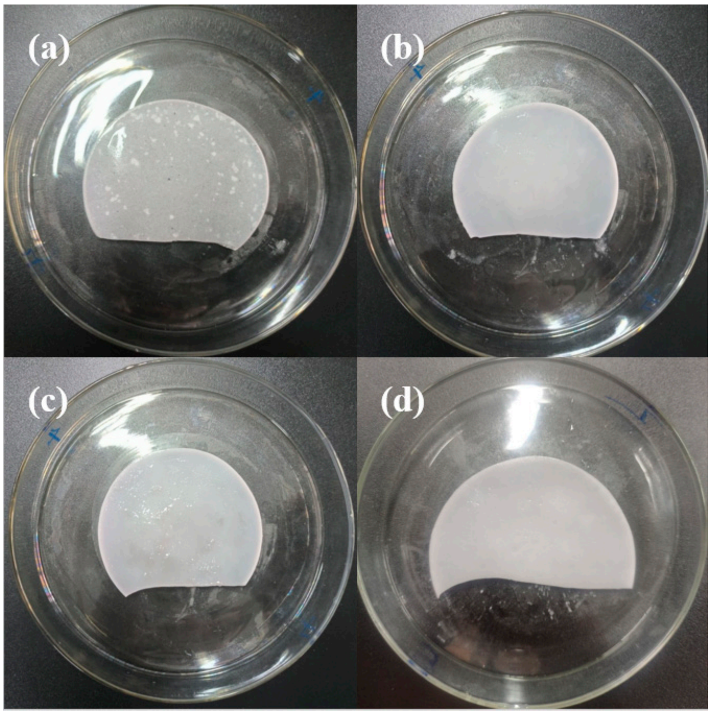


**Figure S6 (a)-(d) Lithium ion sieve hydrogels of PAS-2, PASA-1, PASA-2 and PASA-3.**

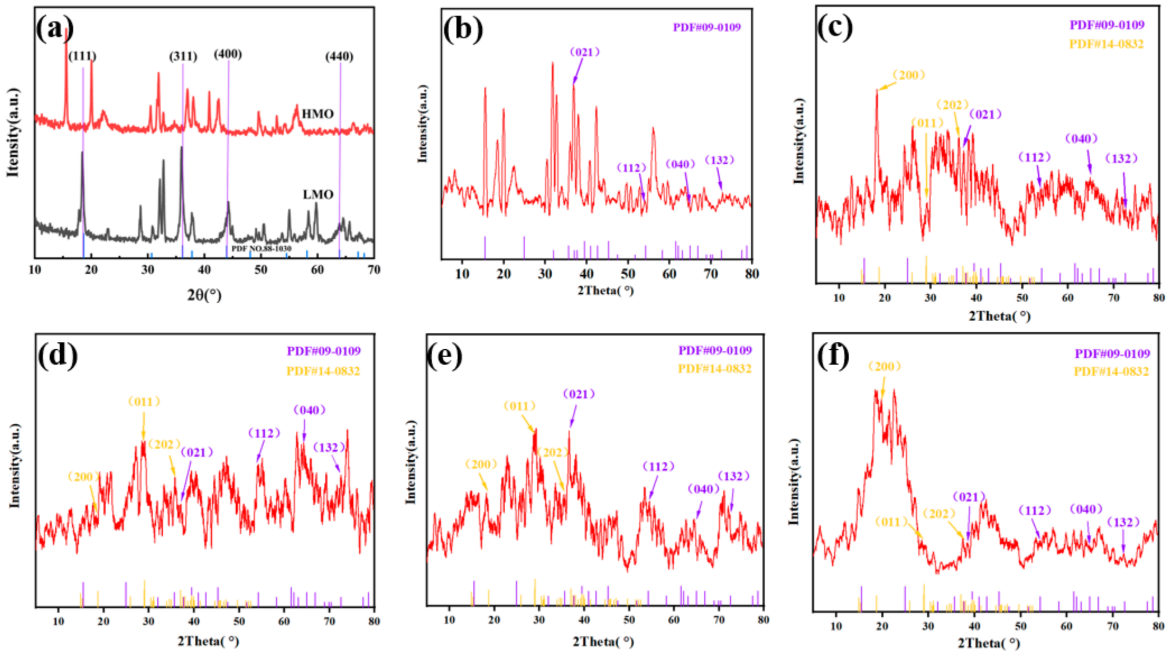


**Figure S7 (a) shows the XRD pattern of LMO; (b) XRD pattern of LIS with lithium-manganese ratio of 1.15; (c)-(f) are XRD patterns of lithium ion sieve hydrogel doped with 0%-3%$Al^{3+}$ respectively.**

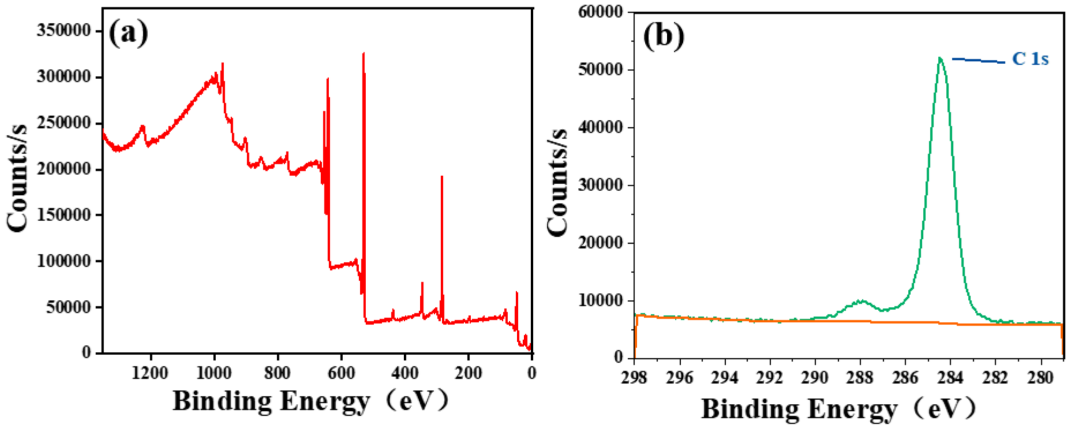


**Figure S8 (a) shows the XPS spectrum of LMAO when the ratio of lithium to manganese is 1.2; (b) XPS spectrum of element C in LMAO.**

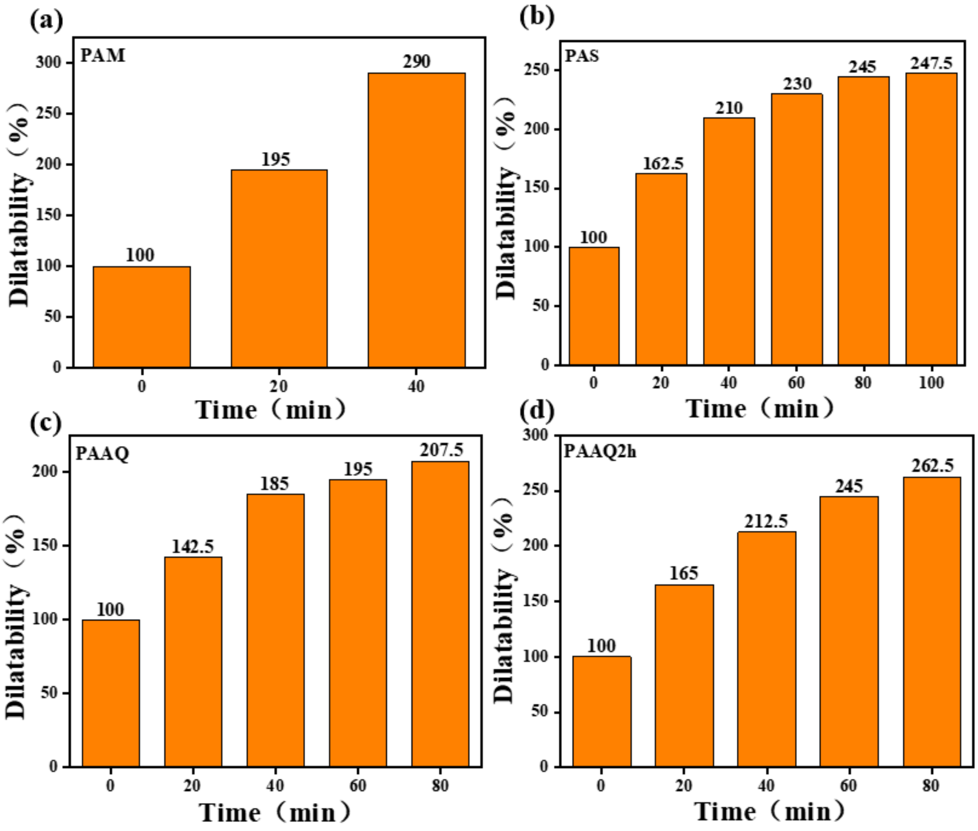


**Figure S9 (a)-(d) show the expansion rate maps of PAM, PAS,PAAQ,PAAQ-2-1 respectively.**
